# Copy-number variation of cancer-gene orthologs is sufficient to induce cancer-like symptoms in *Saccharomyces cerevisiae*

**DOI:** 10.1186/1741-7007-11-24

**Published:** 2013-03-25

**Authors:** Michaela de Clare, Stephen G Oliver

**Affiliations:** 1 Cambridge Systems Biology Centre and Department of Biochemistry, University of Cambridge, Sanger Building, 80 Tennis Court Road, Cambridge CB2 1GA, UK

**Keywords:** Copy-number variation, Model organism, Yeast, Cancer, Haploinsufficiency

## Abstract

**Background:**

Copy-number variation (CNV), rather than complete loss of gene function, is increasingly implicated in human disease. Moreover, gene dosage is recognised as important in tumourigenesis, and there is an increasing realisation that CNVs may not be just symptomatic of the cancerous state but may, in fact, be causative. However, the identification of CNV-related phenotypes for mammalian genes is a slow process, due to the technical difficulty of constructing deletion mutants. Using the genome-wide deletion library for the model eukaryote, *Saccharomyces cerevisiae,* we have identified genes (termed *haploproficient*, HP) which, when one copy is deleted from a diploid cell, result in an increased rate of proliferation. Since haploproficiency under nutrient-sufficient conditions is a novel phenotype, we sought here to characterise a subset of the yeast haploproficient genes which seem particularly relevant to human cancers.

**Results:**

We show that, for a subset of HP genes, heterozygous deletion is sufficient to cause aberrant cell cycling and altered rates of apoptosis, phenotypes associated with cancer in mammalian cells. A majority of these yeast genes are the orthologs of mammalian cancer genes, and hence our studies suggest that CNV of these oncogenic orthologs may be sufficient to lead to tumourigenesis in human cells. Moreover, where not already implicated, this cluster of cancer-like phenotypes in this model eukaryote may be predictive of the involvement in cancer of the mammalian orthologs of these yeast HP genes. Using the yeast set as a model, we show that the response to a range of anti-cancer drugs is strongly dependent on gene dosage, such that intermediate concentrations of the drugs can actually increase a mutant’s growth rate.

**Conclusions:**

The exploitation of data on the phenotypic impact of heterozygosis in *Saccharomyces cerevisiae* has permitted the prediction of CNVs affecting tumourigenesis in humans. Our yeast data also suggest that the identification of CNVs in tumour cells may assist both the selection of anti-cancer drugs and the dosages at which they should be administered if they are to be a beneficial, rather than a deleterious, therapy.

## Background

There is an increasing realisation that copy-number variation, and in particular the loss of one copy of a gene from a diploid cell or organism, can have a significant phenotypic impact. Moreover, the importance of gene dosage in tumourigenesis is becoming increasingly recognised [1], and the widespread aneuploidy and copy-number variations (CNVs) that are the hallmarks of cancer cells are coming to be seen as potentially causative, rather than simply symptomatic [2-4]. Identifying causal CNV-related phenotypes for mammalian genes, however, is hampered by the difficulty in constructing deletion mutants in the higher Eukaryotes, and the fact that homozygous knockout (or complete RNAi knockdown) phenotypes do not necessarily correlate with those elicited by the loss of a single copy of a gene [5,6].

In the model eukaryote *Saccharomyces cerevisiae*, in contrast, high-throughput screens on whole-genome libraries have facilitated the identification of yeast genes for which CNV has a significant effect on cell proliferation. A screen of > 5,800 deletion mutants, each heterozygous for a different protein-encoding gene in the *S*.*cerevisiae* genome, revealed that over 18% of these heterozygotes displayed a significantly reduced growth rate [7]. This haploinsufficient phenotype was displayed even under conditions where there are no external constraints (such as nutrient limitations) on cell growth [8]. For a smaller set (*ca.* 600 genes, listed in Additional file 1: Table S1) of ‘haplo*proficient*’ genes, however, heterozygous deletion of the gene elicits significantly *faster* growth than the wild type. Using these yeast phenotypes, we have previously made correct predictions of a gene-dosage-related phenotype for their orthologous human genes [9], and verified these predictions by controlled RNAi knockdown in human cell lines [10].

The existence of haploproficient genes in the yeast genome indicates that the organism has not evolved to maximize its rate of growth, even when sugars became abundantly available with the emergence of flowering plants approximately 80–100 million years ago [11]. The persistence of these genes in the yeast genome therefore suggests that there must be some major selective advantage that outweighs their growth-rate disadvantages. We demonstrate, here, that this advantage could be the maintenance of genome integrity, which is compromised when the dosage of these genes is reduced. For HP yeast genes, the altered gene dosage not only increases growth rate but, as we will show, copy-number reduction, as opposed to complete gene deletion, is sufficient to result in abnormal progression through the cell cycle, increased accumulation of DNA damage, and altered rates of apoptosis. This set of phenotypes is strongly reminiscent of cancer in mammalian cells. These results complement the recent conclusions from a study of aneuploid yeast strains [12] that the detriment to genome stability is driven by gene-dosage effects (e.g. stoichiometric imbalances), rather than simply by the presence of extra DNA (or additional centromeres; [13]). This emphasises the value of using model organisms to predict which human genes may impact on cancer in a dosage-dependent manner.

The screening of a library of yeast heterozygous deletion mutants for haploinsufficient or -proficient phenotypes is a high-throughput approach to determine what effects quantitative changes in the concentrations of gene products have on phenotype. A related approach is the search for drug-induced haploinsufficiency – in which heterozygous deletion strains exhibit altered sensitivity to a compound as a result of the decrease in the dosage of the target gene. In a pioneering study, [14] exposed a pool of 233 heterozygous mutants to sub-lethal compound concentrations, and later work has successfully elucidated the mode-of-action of novel compounds such as the anti-tumour agent dihydromotuporamine C [15], demonstrating the utility of such genome-wide yeast screens. In the light of our findings on haploproficiency in yeast, we combined both approaches and carried out a screen of anti-cancer agents against a set of *S. cerevisiae* mutants heterozygous for HP genes involved in the DNA damage response-pathway in order to search for altered sensitivities relative to both the WT and the corresponding homozygous (null) deletion mutant. This screen might inform both the appropriate treatment of tumour cells that carry CNVs of the candidate cancer-related genes, and also suggest novel combinations of specific inhibitors which may prove more effect than either drug in isolation.

## Results

### Yeast haploproficient genes are involved in the maintenance of genome integrity and are orthologs of human cancer genes

**Figure 1:**
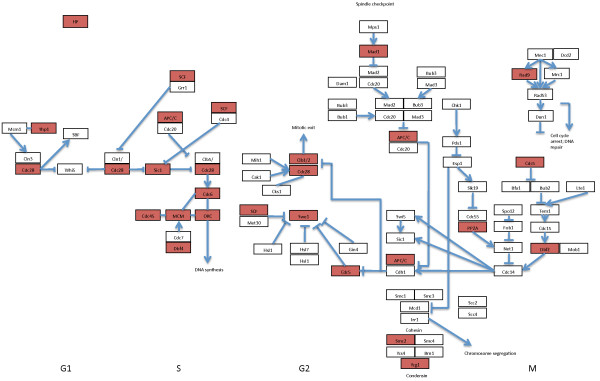
**Haploproficient genes.** The incidence of genes identified in genome-wide deletion library screens [8] as haploproficient within the *S.cerevisiae* cell cycle (haploproficient genes highlighted in red) (pathway diagram derived from the KEGG database [45]). Abbreviations: APC = anaphase promoting complex; SCF = Sck1-Cdc53/CUL-F Box receptor E3 complex; MCM = minichromosome maintenance complex; ORC = origin recognition complex; PP2A = protein phosphatase 2 complex. Haploproficient members of the complexes are listed in Additional file 2: Table S2.

The existence of haploproficient genes, and the inference that the yeast genome has not been optimised for maximal growth rate, does not appear to be an accident, nor unique to *S. cerevisiae.* By examining orthology relationships across the Ascomycetes, we find that haploproficient genes are more highly conserved than the genome average across the lineage (Fischer’s exact test; *p <* 0.01) (Table 1a). Thus, selective pressure has existed toward the *retention* of HP genes for several hundred million years (the evolutionary distance between *S. cerevisiae* and *Sz. pombe*), including through a period of strong selective pressure toward maximizing growth rate that occurred at the time of the whole-genome duplication in the *Saccharomyces* lineage (approximately 100 million years ago [16]). Congruent with our hypothesis of a trade-off between genome stability and growth rate optimisation, we find that haploproficient genes are overrepresented amongst those involved in the maintenance of genome integrity. A Gene Ontology term-enrichment revealed that the HP set is enriched for genes involved in the mitotic cell cycle (*p* = 0.003), and, in particular, the response to and repair of DNA damage (*p =* 0.006) (Figure 1; Additional file 2: Table S2). Given their integral role in maintaining genome stability, it is unsurprising that yeast HP genes are very much more likely to be orthologous to cancer genes than the *S. cerevisaie* genome average (*p* < 10^-10^).

**Table 1:** **a.) *P-*values of enrichment of haploproficient genes amongst those *S.cerevisae *genes having orthologs in Ascomycete species; b.) *P-*values for the enrichment of the subset of *S.cerevisiae *genes involved in the cell cycle**

**Species**	***P-*value of HP gene enrichment amongst orthologs**
*S.paradoxus*	0.02
*S.mikatae*	0.1
*S.bayanus*	0.03
*S.castellii*	0.2
*C.glabrata*	0.07
*S.kluyveri*	0.02
*K.waltii*	0.02
*K.lactis*	0.05
*A.gossypii*	0
*C.albicans*	0.1
*C.tropicalis*	0.08
*C.parapsilosis*	0.04
*L.elongisporus*	0.04
*C.guilliermondii*	0.05
*C.lusitaniae*	0.03
*D.hansenii*	0.07
*A.nidulans*	0.1
*N.crassa*	0.05
*Sz.pombe*	0.06
*Sz.octosporus*	0.05
*Sz.japonicus*	0.02
**Species**	***P-*value of HP cell cycle gene enrichment amongst orthologs**
*S.paradoxus*	0.01
*S.mikatae*	0.001
*S.bayanus*	0.0001
*S.castellii*	0.01
*C.glabrata*	0.01
*S.kluyveri*	0.02
*K.waltii*	0.07
*K.lactis*	0.3
*A.gossypii*	0.001
*C.albicans*	0.9
*C.tropicalis*	0.2
*C.parapsilosis*	0.2
*L.elongisporus*	0.2
*C.guilliermondii*	0.4
*C.lusitaniae*	0.2
*D.hansenii*	0.3
*A.nidulans*	0.6
*N.crassa*	0.7
*Sz.pombe*	0.01
*Sz.octosporus*	0.01
*Sz.japonicus*	0.001

We selected the 30 HP genes (Table 2) involved in the processes of DNA damage repair and sister chromatid segregation as the most relevant candidates in which to examine the relationship between varying gene dosage and cancer-related phenotypes. This ‘HP genome integrity’ (HPGI) set is even more highly conserved than the HP set as a whole (Table 1b); and more likely (than the HP set as a whole) to be orthologous to a cancer gene (*p* < 10^-4^). Nineteen genes in the set have a unique human ortholog (Table 2) and, of these, 12 (*UBX4, IDP1, IDP2, MSH2, RAD1, TOP2, NBP2, MUS81, RAD54, DBF2, STP22, PBS2*) are orthologous to cancer genes as annotated in the Cancer Gene Index (CGI [17]). Table 2 lists the cancer-specific OMIM disease associations of the orthologs of members of the HPGI set.

**Table 2:** Yeast genes comprising the Haploproficient Genome-Integrity (HPGI) set, along with their unique human orthologs, and functional annotations

**Yeast gene**	**Unique human ortholog**	**Human ortholog function & OMIM cancer association (where applicable)**	**Yeast gene product cell cycle-related function**
*BUB2*	-	-	Mitotic exit network regulator; blocks cell cycle progression before anaphase in response to spindle and kinetochore damage
*CCR4*	CNOT6	CCR4-NOT transcription complex, subunit 6	Component of the CCR4-NOT transcriptional complex
*CDC34*	UBE2R2	Ubiquitin-conjugating enzyme E2R 2	Ubiquitin-conjugating enzyme (E2) and catalytic subunit of SCF ubiquitin-protein ligase complex that regulates cell cycle progression by targeting key substrates for degradation
*CHK1*	CHEK1	Serine/threonine kinase and DNA damage checkpoint effector	Serine/threonine kinase and DNA damage checkpoint effector
*CLB1*	CCNB1	Cyclin B1	B-type cyclin involved in cell cycle progression; activates Cdc28p to promote the transition from G2 to M phase
*CTF8*	CHTF8	Subunit of a complex with Ctf18p that shares some subunits with Replication Factor C and is required for sister chromatid cohesion	Subunit of a complex with Ctf18p that shares some subunits with Replication Factor C and is required for sister chromatid cohesion
*DBF2*	-	-	Ser/Thr kinase involved in transcription and stress response; functions as part of a network of genes in exit from mitosis.
*DNL4*	LIG4	DNA ligase required for non-homologous end-joining; OMIM: Lig4 syndrome	DNA ligase required for nonhomologous end-joining (NHEJ)
*IDP1*	IDH1	Isocitrate dehydrogenase 1 (NADP+); OMIM: Oligodendroglioma; piloytic astrocytoma	Mitochondrial NADP-specific isocitrate dehydrogenase, catalyzes the oxidation of isocitrate to alpha-ketoglutarate
*IRR1*	STAG1	Stromal antigen 1	Subunit of the cohesin complex, required for sister chromatid cohesion during mitosis and meiosis
*MAD1*	MAD1L1	Component of the spindle-assembly checkpoint	Coiled-coil protein involved in the spindle-assembly checkpoint; forms a complex with Mad2p
*MEU1*	MTAP	Methylthioadenosine phosphorylase; OMIM: Glioma	Methylthioadenosine phosphorylase
*MLH1*	MLH1	mutL homolog 1, colon cancer, nonpolyposis type 2	Protein required for mismatch repair in mitosis
*MSH2*	MSH2	mutS homolog 2, colon cancer, nonpolyposis type 1; OMIM: colorectal cancer	Protein required for mismatch repair in mitosis
*MUS81*	MUS81	MUS81 endonuclease homolog; OMIM: Bloom syndrome (predisposition to leukemias, lymphomas & carcinomas)	Subunit of the Mms4p-Mus81p endonuclease that cleaves branched DNA; involved in DNA repair, replication fork stability
*RAD1*	ERCC4	Excision repair cross-complementing rodent repair deficiency, complementation group 4; OMIM: Xeroderma pigmentosum (predisposition to basal cell carcinoma & melanoma)	Single-stranded DNA endonuclease (with Rad10p), cleaves single-stranded DNA during nucleotide excision repair and double-strand break repair
*RAD54*	RAD54L	RAD54-like; OMIM: Lymphoma, non-Hodgkin	DNA-dependent ATPase, stimulates strand exchange by modifying the topology of double-stranded DNA; recombinational repair of double-strand breaks in DNA
*RAD61*	-	-	Subunit of a complex (Scc3p, Pds5p, Rad61p) that inhibits sister chromatid cohesion
*RAD9*	RAD9	-	DNA damage-dependent checkpoint protein, required for cell-cycle arrest in G1/S, intra-S, and G2/M; transmits checkpoint signal by activating Rad53p and Chk1p
*SMC2*	SMC2	Structural maintenance of chromosomes 2	Subunit of the condensin complex
*STP22*	TSG101	Tumor susceptibility gene 101; OMIM: Breast cancer	Component of the ESCRT-I complex
*TOP1*	TOP1	Topoisomerase (DNA) I	Topoisomerase I
*TOP2*	TOP2B	Topoisomerase (DNA) II beta 180kDa	Topoisomerase II
*TPD3*	PPP2R1B	Protein phosphatase 2, regulatory subunit A, beta; OMIM: Lung cancer	Regulatory subunit A of the heterotrimeric protein phosphatase 2A (PP2A)
*TUM1*	TST	Thiosulfate sulfurtransferase (rhodanese); OMIM: Tumours	Rhodanese domain sulfur transferase
*UBX4*	ASPSCR1	Alveolar soft part sarcoma chromosome region, candidate 1; OMIM: Alveolar soft-part sarcoma	UBX (ubiquitin regulatory X) domain-containing protein that interacts with Cdc48p
*YCG1*	NCAPG	non-SMC condensin I complex, subunit G	Subunit of the condensin complex
*RIM11*	GSK3A	Glycogen synthase kinase 3 alpha	Protein kinase required for signal transduction during entry into meiosis
*NBP2*	SH3GL1	SH3-domain GRB2-like 1; OMIM: Acute myeloid leukemia	Protein involved in the HOG (high osmolarity glycerol) pathway, contains an SH3 domain that binds Pbs2p
*PBS2*	MAP2K1	MAP kinase kinase	MAP kinase kinase of the HOG signaling pathway; mediates cell cycle arrest

### Orthologs of haploproficient genes exhibit CNV in tumour cells

The haploproficiency phenotype is, by definition, linked to a reduction in gene copy number. Our hypothesis posits that it is copy number variation (specifically, a reduction in gene copy number) of the orthologs of these genes that is relevant to human cancer. To determine the rate of incidence of CNV in orthologs of our HP cell cycle set, we queried our list against the NCI’s Cancer Genome Atlas database (TCGA [17]). No less than 17/19 of the human orthologs of the HPGI set have a copy number log2 ratio of magnitude >0.5 across eight types of cancer. In particular, 12/19 have a copy number reduction (i.e. a log2 ratio of < −0.5) in >25% of patients in one or more of the eight cancer types. This is a significant overrepresentation compared with 7750/~18500 of all genes in TCGA (*p* = 0.07), and with the set of non-HP cell-cycle/DNA repair orthologs (*p =* 0.05). For serous cystadenocarcinoma, kidney renal clear cell, and lung squamous cell carcinomas, there is a significant overrepresentation of orthologs of the HPGI set amongst genes exhibiting frequent copy-number reduction (*p*-values of 2 × 10^-4^, 10^-4^ and 10^-3^ respectively). Moreover, across all eight cancer types, orthologs of the HPGI set are overrepresented with a *p-*value of 0.003 (all corrected for multiple testing).

### CNV of haploproficient genes is sufficient to elicit cancer-like phenotypic responses in yeast

#### Cell cycle phasing

As their growth rate is higher than the previously observed maximum, heterozygous deletion mutants of HP genes must *a priori* progress faster through the cell cycle. Either progress through all the phases of the cycle may be accelerated, or one or more phases must be relatively shorter than in a diploid cell with two copies of the gene. Since the products of HPGI genes are involved in checkpoint controls of the cell cycle, we reasoned that the faster cycle time in the heterozygotes could be the result of cells skipping a compromised checkpoint and therefore progressing faster through that phase of the cycle. This would manifest itself in an altered population distribution between the different cell cycle phases. This same cell cycle dysregulation, arising from compromised tumour suppressor gene function, is a mechanism of oncogenesis in mammalian cells [18]. Previously, we found strong support for the hypothesis that changes in the copy number of the high flux control (HFC) genes alter cell cycle progression in a combined modelling/experimental study of a set of genes involved in the G2/M transition in yeast [10]. This intrinsic link between haploproficiency and cell cycling is also borne out in our finding that copy-number variation of HP genes has no significant effect on yeast chronological lifespan; that is, survival in the extended non-cycling G0 state of stationary phase (see Additional file 3: Table S3).

Using flow cytometry, we analysed the cell cycle progression of heterozygous deletion mutants of the HPGI set, comparing them with the cell cycle profiles of the WT and of the non-cell cycle, non-HP controls *HIS3/his3* and *HO/ho*, non-HP, cell-cycle *HSL1/hsl1*, *CLB2/clb2, CLB5/clb5, CLB6/clb6, CLN2/cln2* and *CLN3/cln3* and the non-cell cycle genes *PNP1, MET7, HRK1* and *TPO3*. To distinguish between the effects of the complete absence of a given gene from those of reducing its copy number from two to one, we also compared the cycle profiles of the heterozygous deletant against that of the diploid homozygous deletants of the same gene. Complete cell cycle profile data is given in Additional file 4: Table S4.

We find that correct dosage of the HPGI genes is essential for normal cell cycling: heterozygous deletion is indeed sufficient to significantly alter the cell cycle profile relative to the WT/non-cell cycle control genes for 13 of the 30 HPGI genes (summarised in Table 3). Typically, the cell cycle perturbation for the heterozygote concurs with that previously reported for haploid null deletants of the gene (as annotated in SGD [19]), however *BUB2* heterozygous deletion confers the opposite phenotype (an decrease in the relative G1 population) to that of both the null diploid and haploid.

**Table 3:** Cell cycle phenotypes of those deletion strains having a cycle profile significantly distinct from the wild-type

**Cell cycle phenotype**	**Genotype**
**G1 Depletion** (>80% of WT, *p <* 0.05)	
	*BUB2/bub2*
	*RAD54/rad54*
	*TPD3/tpd3*
	*mus81/mus81*
	*rad1/rad1*
	*MAD1/mad1*
**S Depletion** (>80% of WT, *p <* 0.05)	
	*RAD9/rad9*
	*rad9/rad9*
	*MUS81/mus81*
	*SMC2/smc2*
	*tpd3/tpd3*
	*TUM1/tum1*
	*YCG1/ycg1*
**G2 Depletion** (>80% of WT, *p <* 0.05)	
	*DBF2/dbf2*
	*MLH1/mlh1*
	*STP22/stp22*

#### Apoptosis rate

In mammalian cells, compromising the link between the DNA damage response and apoptotic pathways can diminish the apoptotic response [20], which is a necessary step *en route* to cancer [21]. Therefore, we carried out tests to determine whether compromising the DNA damage response in yeast by heterozygous deletion of HPGI genes affected the rate of apoptosis (see [22] for a review of *S.cerevisiae* programmed cell death). The degree of apoptosis occurring in the deletion strain populations, in response to treatment with methyl methanesulfonate (MMS) or tert-butyl hydroperoxide (TBHP). was measured by simultaneous annexin V (cell surface phosphatidylserine [23]) and propidium iodide (cell viability) staining [24] to distinguish between apoptotic and necrotic cells. For 15 of the 30 HPGI genes, the heterozygous deletion mutant exhibited a degree of apoptosis significantly different (*p* < 0.05) from than the WT – that is, partial knockdown of the gene expression is sufficient to disrupt the normal apoptotic response of the cell. Whilst exploring the specific mechanisms behind this disruption for each gene is beyond the scope of this paper, it is clear that deviating from the WT dosage of these particular genes has a significant effect on programmed cell death in response to genotoxic agents. Phenotypes for the mutants are described in Table 4; apoptosis rates in *HIS3/his3Δ* and *HO/hoΔ* control strains are comparable to WT.

**Table 4:** Observed apoptotsis-related phenotypes of our HP yeast genes compared with those of their human orthologs

**HP yeast gene**	**Human ortholog**	**Apoptotic phenotype (% dead cells) of yeast heterozygous deletion mutants treated with MMS. (Change in rate of apoptosis, relative to WT, is significant at *p*** **< 0.05 for all yeast heterozygotes.)**		**Mammalian apoptosis-related phenotype from literature**
		**0.01% MMS**	**0.001% MMS**	
*WT*	-	79%	68%	
*CTF8*	CHTF8	49%	-	-
*MSH2*	MSH2	56%	-	RNAi-mediated knockdown decreases apoptosis [25]
*MUS81*	MUS81	56%	-	-
*TPD3*	PPP2R1B	60%	-	RNAi-mediated knockdown decreases apoptosis [26]
*TOP1*	TOP1	55%	-	RNAi-mediated knockdown reduces nuclear apoptosis [27]
*RAD54*	RAD54L	44%		-
*SMC3*	SMC3	-	30%	RNAi-mediated knockdown increases apoptosis [28]
*RAD1*	ERCC4	-	22%	-
*DNL4*	LIG4	-	46%	Extensive apoptosis in mouse deletion homozygotes [29]
*MAD1*	MAD1L1	-	20%	-
*MEU1*	MTAP	-	94%	Pharmacological targeting of MTAP promotes apoptosis [30]
*MLH1*	MLH1	-	93%	Abnormal apoptosis (OMIM)
*CHK1*	CHEK1	-	82%	Blocking CHK1 induces apoptosis [31]
*UBX4*	ASPSCR1	-	80%	Regulator of apoptosis (OMIM)

The phenotypes observed for the HPGI heterozygous deletants were compared with those reported in the literature for the RNAi-mediated knockdown or pharmacological inhibition of their mammalian orthologs. The congruence between the yeast and mammalian phenotypes is summarized in Table 4. For 2/4 cases of increased apoptosis upon copy-number reduction, the phenotype is also observed in human cells, however for *MLH1*/MLH1 and *UBX4/*ASPSCR1 the relationship between gene dosage and apoptosis in human cells is acknowledged, but unclear [32].

Similarly, for 4/11 genes prompting reduced apoptosis upon copy-number reduction, knockdown of the human ortholog has been reported to cause a similar phenotype; we could find no report for the orthologs of a further 5 of the 11. For *SMC3*/SMC3 and *DNL4/*LIG4, however, copy-number reduction in yeast reduced apoptosis, whilst knockdown in mammalian systems has been reported to increase its occurrence [28,29]. However, both of these reports involve a complete deletion of the gene, rather than the heterozygous deletion in which we observed the yeast phenotype. This suggests that further investigation in human cells of the effects of varying gene dosage of these genes, in particular, would be worthwhile.

#### Haploproficiency & cancer drug sensitivity

Given the strong connection that we have observed between yeast haploproficiency and human cancer, it is unsurprising that the human orthologs of many of the HPGI genes are the targets of various anti-cancer compounds (both approved drugs and those in late-stage clinical trials; see Table 5). However, in the light of the *increased* growth upon reduction of HP gene dosage, and the dosage-specific phenotypes reported above, it is possible that inhibitor treatment of a tumour cell could elicit the opposite to the desired response – i.e. increased proliferation rather than cell death – if complete ablation of the HP target function is not achieved. Therefore, we examined the drug sensitivity of wild-type yeast, and the heterozygous and homozygous deletion mutants for each of the non-essential HPGI genes. We also included the deletion mutants for an additional five yeast genes (*HRK1, TPO3, MET7, PNP1* and *GPD1*), which are HP and whose products are orthologous to specific cancer-drug targets.

**Table 5:** Anticancer drugs and the hypertolerant phenotype of yeast strains heterozygous for the HP gene encoding their target protein

**Drug**	**Concentration in solid medium (uM)**	**Concentration in liquid medium (uM)**	**Mode of action**	**Specific HP genetic target (yeast/human)**	**Hyper-tolerant yeast strains**	**Cancer in which CNV-reduction of the gene deleted** **in the hypertolerant strain occurs**
				RAD54/RAD54L		
Cycloheximide	0.36, 1.8	0.1	Inhibition of translation	RIM11/GSK3B	*IRR1/irr1*	-
Mitoxantrone	10, 25	-	TOPII inhibition	TOP2/TOP2A	-	-
					*NBP2/nbp2*	
					*GPD1/gpd1*	Serous adenocarcinoma (*NBP2)*
Methotrexate	200	2, 100	Inhibition of nucleoside and purine synthesis	MET7/FPGS	*RIM11/rim11*	Renal cell carcinoma *(GPD1)*
Phleomycin	1, 2	0.1, 0.5	DNA strand break induction	-	*MET7/met7*	Serous adenocarcinoma
5-fluorouracil	200	2, 100	Inhibition of pyrimidine synthesis	-	*IDP1/idp1*	-
Tamoxifen	25	-	Estrogen receptor antagonist	-	-	-
					*NBP2/nbp2*	
Aminopterin	10	0.1,1, 5, 10, 50, 100	Inhibition of DNA, RNA & protein synthesis	-	*TOP1/top1*	Serous adenocarcinoma
SAHA/vorinostat	10, 20	-	Histone deacetylase inhibition	RPD3/HDAC2	-	-
Bay 11-7085	20, 40	-	NF-kB TF inhibitior; proapoptotic agent	-	-	-
Cantharidin	30, 60	6, 30	Inhibition of PP2A	TPD3	*UBX4/ubx4*	-
PD98059 (2'-amino-3'-methylflavone)	40, 80	4	MEK1 MAPK inhibition	PBS2/MAP2K1	*NBP2/nbp2*	Serous adenocarcinoma
					*HRK1/hrk1*	
					*MLH1/mlh1*	
Hydroxyurea	100000, 200000	100, 1000	Inhibition of DNA synthesis; DSB induction	RAD54/RAD54L	*STP22/stp22*	Renal cell carcinoma (*MLH1)*
Lithium chloride		-	Alternative RIM11 inhibitor	RIM11/GSK3B	-	
Caffeine	1000, 5000	-	Alternative RAD54 inhibitor	RAD54/RAD54L	-	
Methyl methanesulfonate		-	DNA methylation, causing DSB or stalled replication forks	-	-	
DPI	10, 20	-	NAD(P)H oxidase inhibition	-	-	
Tert-butyl hydroperoxide (TBHP)		-	Free-radical generation	-	-	
Clotrimazole	2.5, 5, 7.5	-	Antifungal; increases cell wall permeability	-	-	

Where the human ortholog of a yeast HPGI gene product is a cancer-drug target (Table 5) we included in our screen either the commercial drug (where available) or alternative inhibitors (for example, PD98059 for Pbs2p). In total, 18 drugs were screened (see Table 5): two of these were specific alternative inhibitors; 15 were compounds either approved for human cancer treatment or shown to be effective in cell culture; and one control treatment of clotrimazole, a fungicide not known to have anti-cancer properties.

**Figure 2:**
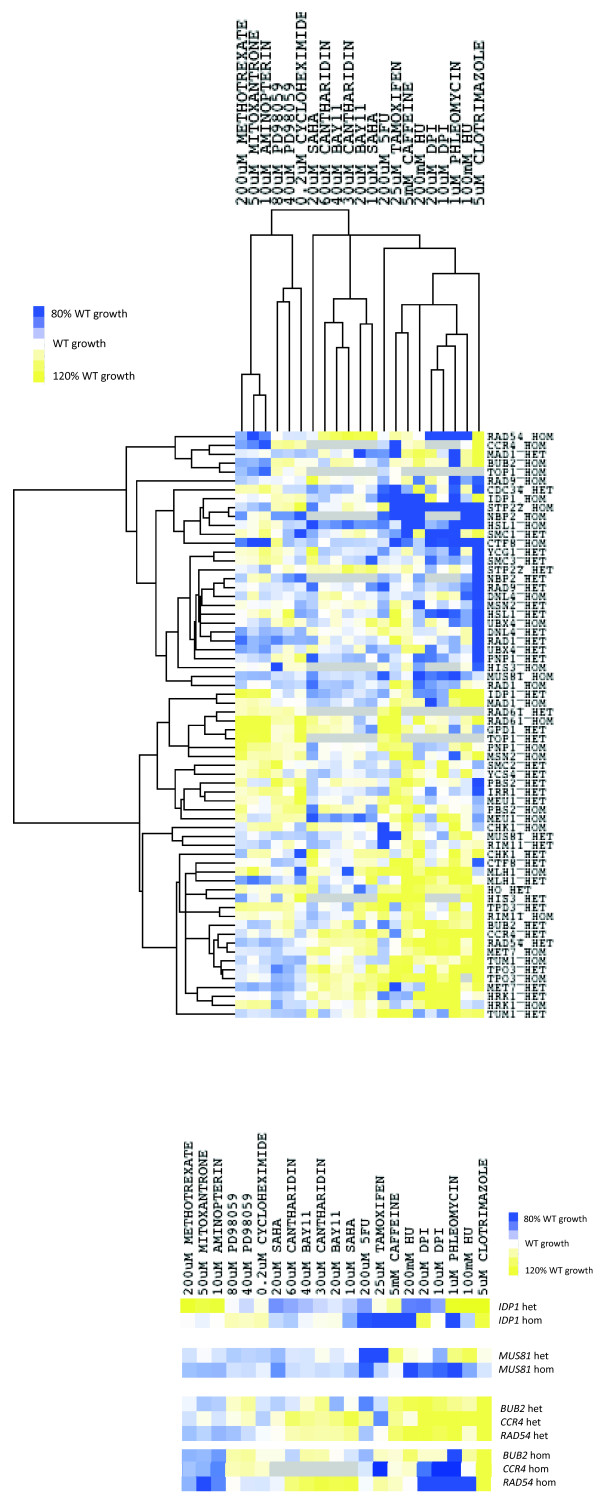
**Interrelationships of drug-response profiles of yeast deletion mutants. Left panel:** Hierarchical clustering of the response of the heterozygous and homozygous deletion mutants of the HPGI set to treatment with a panel of anti-cancer compounds. **Right panel:** Comparison of the drug-response profiles for the heterozygous and homozygous mutants of those HPGI genes for which cancer drug sensitivity differs markedly according to gene copy number.

Drug sensitivity profiles were clustered (Figure 2 left panel) by agglomerative hierarchical clustering [33]. The profile elicited by a compound can reveal its mode of action, and thus similarities between drug profiles may indicate the targeting of the same pathways in yeast. Reassuringly, known modes of action are reflected in our profiles – for example, the sensitivity of cell wall integrity/morphogenesis deletion mutants to phleomycin (Figure 2 left panel), which causes cell wall lesions [34].

Control (non-cancer drug) clotrimazole treatments are reasonably distinct from the cancer drug treatments. Aminopterin and methotrexate (which share a common, antifolate, mechanism of action) PD98059, cycloheximide and mitoxantrone cluster together, driven largely by the resistant phenotypes of deletion mutants of genes involved in chromosome condensation and segregation (*SMC2*, *TOP1*, *RAD61*, *IRR1*, *MAD1*). A second cluster is formed by Bay11, cantharidin and vorinostat (SAHA), all of which act as inhibitors of the NF-κB pathway in mammalian cells [35-37]. This pathway is absent in *S.cerevisiae*[38] however, the clustering of the compounds suggests that their modes of action, though distinct from the mammalian-cell pathway, may also be related in yeast. *rad54/rad54* mutants are particularly resistant to all three drugs, where, in general, these mutants are sensitive to all other treatments. Vorinostat has been demonstrated to induce DSBs in acute myeloid leukemia cells [39], suggesting that the DSB-repair function of Rad54p could potentially be a target. Indeed, the *RAD54* ortholog, RAD54L, is strongly down-regulated in LNCAP (prostate adenocarcinoma) cells treated with vorinostat [40]. This is also the case for the mammalian orthologs of *CHK1* and *TOP2*, which are also resistant (though less strongly so) to the vorinostat /Bay11/cantharidin cluster. We have since demonstrated [10] that the *magnitude* of these drug-response phenotypes in yeast (deviations of up to ±20% of WT growth rate) is sufficient to translate into observable phenotypes in tumour cell lines.

#### Targeting the CNV profile of tumour cells

**Figure 3:**
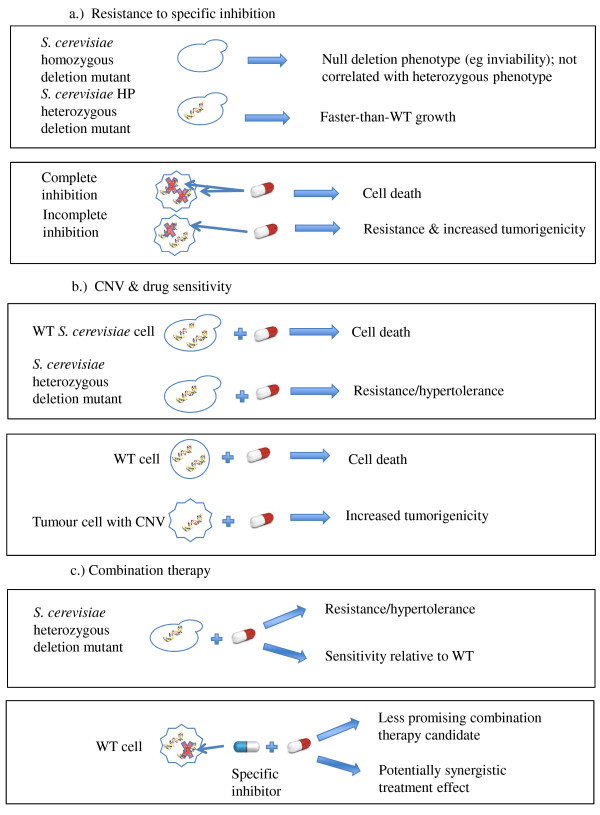
**The relevance of haploproficiency and hypertolerance to drug interventions. a**) specific inhibition, but not complete protein-product ablation, of an HP target prompts increased growth; **b**) case where the cancer cell itself bears a CNV of an HP gene, the deletion mutant phenotype may better predict cancer cell drug response; **c**) case where the deletion mutant for a specific gene is hypertolerant to a compound inhibiting a distinct target, a combination therapy of the same compound plus an inhibitor of the first gene is unlikely to elicit a positive therapeutic response.

Where a copy of a gene is frequently lost in tumour cells, as is the case for orthologs of the HPGI set, the phenotype of the heterozygous deletion mutant of the yeast ortholog might better reflect the tumour cell’s response to drug treatment (Figure 3b). For a significant number of homozygous/heterozygous deletant pairs (9/30, *p* = 0.01) the drug sensitivity phenotypes are quantitatively dissimilar (by Euclidean squared distance, i.e. diverging at the top level of branching) (Figures 2 left and right panel). For these genes, therefore, gene dosage may be highly relevant to pharmaceutical efficacy. These eight genes (*CCR4, TOP1, IDP1, RAD54, BUB2, MUS81, PNP1,* and *MAD1*) have, on average, a greater number of protein-protein interactors (as annotated in BioGrid [41]) than the average for our HPGI set as a whole (180 unique interactors versus 135). This suggests that gene dosage balance may be an important contributor to phenotype for genes which are more central in interaction networks. We have explored the relationship between gene copy number and drug response further in [8].

Previously, we reported the striking result that treatment of wild-type yeast with low concentrations of inhibitors targeting HP genes can induce the same increased proliferative phenotype as is observed upon reducing the copy number of the HP target, a phenomenon we termed *hypertolerance*[8]. We observe this phenotype again for treatment with cantharidin, which inhibits the PP2A complex, the subunits of which are strongly haploproficient (Additional file 5: Table S5), but not for any of the general cytotoxic compounds in the drug screen, reinforcing our previously-established link between the specific inhibition of a haploproficient target and drug hypertolerance. A number of heterozygous strains also exhibited hypertolerance in solid-media screens, which was subsequently confirmed by titrating across a range of drug concentrations in liquid medium (Table 5). For tumour cells bearing the orthologous CNVs, this would suggest a contraindication for treatment with the particular drug. In particular, the *NBP2/nbp2* heterozygote is multiply drug-hypertolerant, of note since its ortholog *SH3GL1* frequently has reduced copy-number in serous cystadenocarcinoma.

Conversely, for *PNP1*, the heterozygotes are mitoxantrone/methotrexate/aminopterin and vorinostat/Bay11/cantharidin *sensitive*, and the homozygotes resistant. This phenotype may be a instance of ‘obligate haploinsufficiency’ [42], whereby fitness is compromised as the gene dosage is reduced below some threshold value, but the phenotype is subsequently rescued through the switching on of a compensatory pathway.

## Discussion

The data presented here for the *S.cerevisiae* HPGI set point to the striking result that copy-number variation (in this case, deletion of just one gene copy from a diploid cell), rather than complete gene loss, is sufficient to elicit deleterious phenotypes, in particular those reminiscent of cancer in mammalian cells. This concurs with the increasing appreciation of the significance of copy-number variation to the genesis and progression of disease. In particular, there is a general lack of significant correlation of phenotypes between the heterozygous and homozygous (null) deletion mutants of a given gene. Nor are the phenotypes of the null mutants necessarily more extreme than those of the heterozygous deletants, as might naively be expected from a linear ‘dosage hypothesis’ [43]. This emphasises the need to systematically investigate the phenotypic impact of gene dosage, rather than relying on knockout, or total-knockdown, studies.

A mechanism by which heterozygous deletion of a gene could be just as disruptive as a complete knockout is via the disruption of the stoichiometric ratios of the sub-units of protein complexes. This has been proposed as a mechanism for haploinsufficiency in the 'balance hypothesis' of Papp *et al.*[44-46]. It also suggests that dosage compensation, in which the impact of the heterozygous deletion of a gene is mitigated by the increased expression of the remaining copy, does not play a significant role amongst our HPGI gene set. Indeed, [47] showed that such dosage compensation is, in general, rare in *S.cerevisae*, and very few HP genes are amongst those exhibiting significant compensation.

For cell cycle phasing, heterozygous gene deletion is often sufficient to perturb the cycle from the WT profile, which indicates that cell cycle proteins are required in yeast at greater than 50% of WT dosage (the quantitative relationship between gene copy number and cell cycling is further explored in [10]). Similarly, heterozygous deletion of the yeast ortholog is, in general, both sufficient to alter the degree of apoptosis occurring in response to DNA-damaging agents (for 15 out of 30 HPGI genes), and to elicit the same apoptotic phenotype (in 8 out of 15 cases) as is reported for complete protein-product knockdown in mammalian cells. This general conservation of apoptotic phenotypes suggests that the DNA damage-related pathway(s) controlling apoptosis in *S.cerevisiae* are similar to those in mammals. This could be investigated further by undertaking a global survey of the effect of gene deletion on apoptosis rates in yeast, particularly since the current data on the degree of apoptosis in *S.cerevisiae* is limited (SGD, for example, reports altered apoptotic phenotypes for only 37 null/conditional mutants and 13 overexpression strains, none of which are in the HPGI set). In four cases, an altered apoptosis rate was observed in the yeast deletion mutants but has not, so far, been reported for the RNAi knock-downs of their human orthologs. We would suggest that, based on the apparent predictive value of the yeast phenotype, these genes should become the focus of an RNAi study in mammalian cells.

### HP genes and the efficacy of anti-cancer drugs

This critical importance of considering gene dosage in the context of pharmaceutical intervention in cancer is further emphasised in the copy-number-dependence of drug sensitivity phenotypes that we observed with our yeast model. The mechanisms by which copy-number variation may exert a significant effect on phenotype are summarised in Figure 3.

Firstly, products of the mammalian orthologs of several of the yeast HPGI set are the targets of specific drugs used, or proposed for use, in treating cancers. Our data suggest that, in a majority of such cases, complete inhibition of the activity of a target protein product is necessary to achieve a positive therapeutic outcome. Incomplete inhibition, analogous to heterozygous deletion of the gene encoding the target, prompts increased proliferation or hypertolerance – the opposite of the intended anti-cancer effect. Furthermore, even if a given gene is not the intended specific target of a chemotherapeutic treatment, the modes-of-action, and secondary impacts, of many cancer drugs have not been fully elucidated. Drug-induced haploinsufficiency (or haplotolerance) data from yeast can contribute to a better understanding of both drug mechanisms and the functional conservation of drug-metabolism pathways between yeast and humans. For example, the clustering of mutant phenotypes in response to the mammalian-NF-κB inhibitors in this study suggests that all three compounds act through a common pathway in *S.cerevisi*ae, which may be mediated by Rad54p.

Secondly, we observed that varying the copy number of the yeast ortholog can significantly alter the phenotypic response to drug treatment. This is especially for genes whose human orthologs have a high likelihood of CNV in tumour cells, In particular, several deletion strains are either more resistant than the WT, or themselves hypertolerant in response to a specific drug treatment. The impact of specific CNVs on drug sensitivity is becoming increasingly appreciated, and we believe our yeast mutant approach represents a high-throughput complement to the creation of drug-CNV profile ‘fingerprints’ for tumour cells (see e.g. [48]), and a model for determining the most effective drug interventions for tumours with a particular CNV profile.

Lastly, our data on the sensitivity/resistance of deletion mutants could potentially inform the design of combination therapies. Where a particular (heterozygous) deletion mutant is sensitive to a given drug, a combination therapy consisting of that drug plus an inhibitor of the product of the heterozygous gene could have a synergistic effect in reducing cell proliferation. Conversely, considering pairs of genes/drugs for which the deletion mutant is resistant to treatment could assist in identifying those drug combinations which are unlikely to succeed, reducing the search space involved in an otherwise expensive combinatorial problem [49].

Finally, the several instances in which we observed that the heterozygous deletion mutant is more sensitive to drug treatment than the homozygote provide evidence for the ‘obligate haploinsufficiency’ hypothesis of Berger *et al.*[42]. Identifying such genes, and defining the protein product threshold below which their associated compensatory pathways are activated, could help to define the precise inhibitor dosage to achieve maximal therapeutic efficacy.

## Conclusions

Haploproficient (HP) genes and are for which, when one copy is deleted from a diploid cell, the rate of cell proliferation is increased. We have demonstrated that, for a subset of yeast HP genes, heterozygous deletion causes aberrant cell cycling and altered rates of apoptosis, which are phenotypes associated with cancer in mammalian cells. A majority of these yeast genes are the orthologs of mammalian cancer genes, and hence our studies suggest that CNV of these oncogenic orthologs may be sufficient to lead to tumourigenesis in human cells. Moreover, where not already implicated, this cluster of cancer-like phenotypes in this model eukaryote may be predictive of the involvement in cancer of the mammalian orthologs of these yeast HP genes. Using the yeast set as a model, we have shown that the response of the heterozygotes to a range of anti-cancer drugs is strongly dependent on gene dosage, such that intermediate concentrations of the drugs can actually increase a mutant’s growth rate. This suggests such compounds may need to achieve near-total inhibition of target activity if they are to be a beneficial, rather than a deleterious, therapy.

## Methods

### Haploproficient genes and orthology analysis

The set of *S.cerevisiae* genes which are haploproficient in turbidostat culture was obtained using the growth data of [8] and an FDR cutoff of 0.02. This stringent FDR cut-off rigorously defines those genes for which heterozygosity confers a strong fitness advantage, but has no effect on the functional enrichment of genes identified as haploproficient. Genes defined as ‘haploproficient’ for the purposes of this study are listed in Additional file 1: Table S1. The set of chromosome maintenance-associated HP genes described in [8] overlaps, but is not coincident, with the HPGI set studied here, since the current set also includes DNA damage-response genes.

Orthology assignments were made using the InParanoid algorithm [50] and compared with the results of a BLAST [51] reciprocal best-hits search. GO enrichment searches were performed using the Babelomics 4 FatiGO tool [52]. To assess the significance of HP gene conservation, the number of HP genes having orthologs in a given Ascomycete species, given the number of *S. cerevisiae* HP genes, was compared against the whole-genome conserved proportion using a χ^2^ or Fisher exact test (depending on sample size), with the null hypothesis of identical distribution. All findings of significance were reiterated using a Z test for difference of proportions. Where necessary, *P* values were corrected for multiple testing using the Bonferroni correction. Cell cycle and DNA damage repair pathways were obtained from the KEGG pathway database [53].

Expression data for *S.cerevisae* genes was obtained from the Saccharomyces Genome Database [54]; and protein expression levels from [55]. A list of human cancer genes/oncogenes was obtained from the Cancer Gene Index [17]; enrichment of HP genes amongst the orthologs was determined using a χ^2^ test as above. CNV incidence across eight tumour types (breast invasive carcinoma, rectum adencarcinoma colon adenocarcinoma, kidney renal cell clear carcinoma, uterine corpus endometrioid carcinoma, glioblastoma multiforme, acute myeloid leukemia, lung adenocarcinoma, lung squamous cell carcinoma, serous cystadenocarcinoma) as measured by comparative genomic hybridisation, was obtained from the NCI Cancer Genome Atlas online data browser [17] with a copy number (log2 ratio) of magnitude >0.5 taken as the significance threshold. Details of the sampling and analysis of the tumour samples are described in [17]. A *P-*value for HP ortholog overrepresentation was calculated using a χ^2^ test .The TGCA database was also used to perform a pathway search for overrepresentation of HP orthologs.

### Yeast strains

In total, 30 HP genes were chosen for analysis, based upon the criteria discussed in the Results above. The heterozygous deletion mutant of each gene was obtained from the heterozygous diploid deletion library (Open Biosystems), in the BY4743 (*MAT****a****/α, his3D1/his3D1, leu2D0/leu2D0, LYS2/lys2D0, met15D0/MET15, ura3D0/ura3D0*) genetic background. For non-essential genes, the homozygous deletant was retrieved from the analogous homozygous diploid deletion library (Open Biosystems).

Control strains were the BY4743 WT, along with the heterozygous deletion mutant of the non-functional *his3* locus; the non-HP, non-cell cycle *ho*/*HO* heterozygous deletion strain; and the heterozygous deletion mutant of the non-HP, cell cycle gene *HSL1.* In addition, heterozygous deletion mutants of the G1 and G2 cyclins were included in several of the experiments. A complete list of the strains used is provided in Additional file 6: Table S6.

### Cell-cycle profiling

Flow cytometric analysis of the deletion strains’ cell cycle profiles was carried about following the method of [56]. Briefly, ~10^7^ cells in mid-exponential phase were harvested, washed, and fixed in absolute ethanol at 4C overnight. Fixed cells were then collected, washed, and boiled for 15 minutes in 2 mg/mL RNAse in 50 mM Tris-Cl (pH 8), and incubated at 37C for 2–12 hours. Cells were resuspended in protease solution (5 mg/mL pepsin, 4.5 μL/mL concentrated HCl), incubated for 15 minutes at 37C and resuspended in 50 mM Tris (pH 7.5). For analysis, 50 mL of cell suspension was added to 1 mL of 1 mM Sytox Green in 50 mM Tris pH 7.5), vortexed and analysed using a Cyan flow cytometer (Beckman Coulter). FlowJo (Tree Star) analysis software was used to fit histograms to the peaks representing 1C and 2C DNA content, and thereby calculate the number of cells in the G1 and G2 phases, and infer the number in S phase from the remaining fraction of the population.

### Chronological lifespan assay

Cultures were inoculated from frozen stocks, grown overnight in YPD at 3°C, and 200mL of each was transferred into a well of a 96-well microtiter plate (Corning). Strains were present in duplicate on each plate, with a buffer of WT in the wells around the edge of the plate, so edge effects would not impact test colony measurements. A Singer Rotor HDA colony pinning robot was used to spot four replicates of each well onto a YPD + 10 μg/mL phloxine B (Sigma) plate. Phloxine B is a fluorescein derivative taken up when the cell membrane is disrupted upon cell death [57]. Plates were incubated for 48 hours at 3°C and photographed using an Epson 1240 Scanner. The colony images were analysed using a custom image-analysis code written in MatLab, with colony size measured by pixel count, and fraction of dead cells by the intensity of colony redness [10]. Since these parameters are independent, this allowed the dissection of the effect of cell viability upon colony growth from that of growth rate variation. The 96-well liquid cultures were incubated at 3°C, and, every second day over a period of three weeks, the colony-pinning onto YPD + phloxine B and image analysis repeated. For each plate, the median culture intensity for each strain was compared with the growth of the WT on that plate, and also with the strain growth and viability after the initial 48-hour period. The experiment was performed twice.

At several points throughout the 3-week period, several strains were selected at random, and viability assayed by performing serial dilutions and counting colony-forming units. These results were checked for compatibility with the microplate viability results.

### Apoptosis assays

The rate of occurrence of apoptosis in the different strain populations was measured in two ways. Apoptosis was first induced by pretreating cells with 0.001%, 0.01% MMS, 0.0001% or 0.001% TBHP in overnight culture; keeping a negative, non-induced WT control sample.

The translocation of phosphatidyl serine to the cell surface, a marker of apoptosis [58], was measured using an Annexin V-FITC Apoptosis Detection kit. (Sigma). Cells were harvested, washed in 1.2M sorbitol, 0.5 mM MgCl_2_, 35 mM K phosphate (pH 6.8) and then digested in 5.5% glusulase (Sigma) and 15 U/mL lyticase (Sigma) for 2 hours at 28C. Spheroplasts were harvested, washed in binding buffer (10 mM Hepes/NaOH pH 7.4, 140 mM NaCl, 2.5 mM CaCl_2_ in 1.2 M sorbitol buffer) and resuspended in binding buffer/sorbitol. 5 mL of FITC-labelled annexin V, and 10 mL of 10010 mg/mL propidium iodide were added to each sample, with control samples containing 1.) no label, 2.) FITC-annexin V only, and 3.) PI only. Fluorescence was quantified using a CyAn (Beckman Coulter). Gates were fitted on the basis of the the control samples, dividing a log PI versus log FITC plot into four quadrants: lower left (neither FITC nor PI-stained) – viable cells; upper left (PI stain only) – necrotic cells; lower right (FITC only) – early apoptotic cells; and upper right (PI and FITC-stained) – late apoptotic cells. FlowJo software (TreeStar) was used to count the fraction of the total cell population in each quadrant. The proportion of both necrotic and apoptotic cells for each strain was normalised to strain viability (i.e. on the basis of the proportion of cells assigned to the lower-left FITC/PI quadrant), and the ratio of necrotic:apoptotic cells calculated. Ratios for each strain were normalised to the WT value, and the standard deviation across all samples calculated. Strains having a necrosis:apoptosis ratio further than 1.5x this standard deviation from WT levels were deemed to demonstrate abnormal apoptosis rates.

### Growth rate and drug sensitivity assays

Growth and drug sensitivity assays were performed both on solid media and in liquid cultures. For solid assays, the required drug concentration was added to YPD-agar containing 10μg/m/mL phloxine B. Overnight cultures of the strains were spotted onto the (drug-containing) plates using a Singer rotor, as above. Plates were incubated at 3°C and photographed at 24 and 48 hours and analysed using an image-processing code as described above. Strain growth and viability was compared both with WT growth on the same plate, and with growth on YPD-agar (or YPD-agar plus DMSO, where the drug is DMSO-soluble). The ratio of viability and size with and without drug was calculated for every strain on a plate, and the standard deviation of all ratios calculated. Strains having a drug:untreated ratio greater than or less than two standard deviations from that of the WT were deemed to be resistant and sensitive, respectively.

Assays in liquid culture were performed by transferring 5mL of overnight culture into each well of a 96-well microtitre plate, containing 200 μL of YPD plus the required concentration of drug. Absorbance was measured for 30 hours at 3°C using a BMG Optima platereader, maximum growth rate calculated using a curve-fitting script written in R, and the growth rate for each strain compared with that of the WT in the same plate, and growth in YPD/YPD + DMSO.

## Abbreviations

CNV: Copy number variation; HP: Haploproficient; HPGI: Haploproficient genome integrity; TBHP: Tert-butyl hydroperoxide; MMS: Methyl methanesulfonate; SGD: Saccharomyces Genome Database.

## Competing interests

The authors declare that they have no competing interests.

## Authors’ contributions

MdC and SGO conceived the study together. MdC performed the experiments and analysed the data. Both authors wrote the paper. Both authors read and approved the final manuscript.

## Supplementary Material

Additional file 1: Table S1 List of *S.cerevisiae* haploproficient genes. Click here for file

Additional file 2: Table S2 Haploproficient members of the major complexes within the *S.cerevisiae* cell cycle. Click here for file

Additional file 3: Table S3 Chronological lifespan data for heterozygous deletion mutants of the HPGI set compared with control strains. Click here for file

Additional file 4: Table S4 Cell cycle profiling data for the heterozygous and homozygous deletion mutants of the HPGI set, and control strains. Click here for file

Additional file 5: Table S5 Growth phenotypes of heterozygous deletion mutants of genes encoding subunits of the yeast PP2A complex. Click here for file

Additional file 6: Table S6 Yeast strains used in this study. Click here for file
