# Supplementary material for: Copy-number variation of cancer-gene orthologs is sufficient to induce cancer-like symptoms in Saccharomyces cerevisiae
Source: BMC Biol. 2013 Mar 25;11:24. doi: 10.1186/1741-7007-11-24 (PMC3635878; doi:10.1186/1741-7007-11-24)
Supplement: Additional file 2: Table S2 — Haploproficient members of the major complexes within the S.cerevisiae cell cycle. [file 1741-7007-11-24-S2.docx]

**Additional Table 2:** Haploproficient members of complexes involved in the *S.cerevisiae* cell cycle, and the non-homologous end joining (NHEJ) and mismatch repair (MMR) DNA damage repair pathways.

| Pathway/Complex | HP subunits |
| --- | --- |
| PP2A (protein phosphatase 2A) | RTS1 |
|  | PPH21 |
|  | PPH22 |
|  | TPD3 |
| SCF | CDC4 |
| APC/C (anaphase promoting complex) | APC1 |
|  | APC9 |
| MCM (minichromosome maintenance complex) | MCM6 |
| NHEJ (non-homologous end joining) | MRE11 |
|  | DNL4 |
|  | LIF1 |
|  | POL4 |
| MMR (mismatch repair) | PMS2 |
|  | MLH1 |
|  | MSH2 |
