# Supplementary material for: Copy-number variation of cancer-gene orthologs is sufficient to induce cancer-like symptoms in Saccharomyces cerevisiae
Source: BMC Biol. 2013 Mar 25;11:24. doi: 10.1186/1741-7007-11-24 (PMC3635878; doi:10.1186/1741-7007-11-24)
Supplement: Additional file 5: Table S5 — Growth phenotypes of heterozygous deletion mutants of genes encoding subunits of the yeast PP2A complex. [file 1741-7007-11-24-S5.docx]

**Additional Table 5:** Growth phenotypes of heterozygous deletion mutants of genes encoding subunits of the yeast PP2A complex.

| **Gene** | **Heterozygote growth phenotype in turbidostat** | **Heterozygote growth rate relative to WT in batch culture** |
| --- | --- | --- |
| *PPH21* | HP | 1.21 |
| *PPH22* | HP | 1.1 |
| *RRD1* | NOT HFC | 0.99 |
| *RRD2* | NOT HFC | 1.01 |
| *RTS1* | HP | 1.07 |
| *TPD3* | HP | 1.19 |
| *TAP42 (binding partner)* | HP | - |
| *CDC55* | Not in deletion mutant collection | - |
