# Supplementary material for: Copy-number variation of cancer-gene orthologs is sufficient to induce cancer-like symptoms in Saccharomyces cerevisiae
Source: BMC Biol. 2013 Mar 25;11:24. doi: 10.1186/1741-7007-11-24 (PMC3635878; doi:10.1186/1741-7007-11-24)
Supplement: Additional file 6: Table S6 — Yeast strains used in this study. [file 1741-7007-11-24-S6.docx]

| **Strain** | **Genotype** |
| --- | --- |
| TPD3 heterozygous deletant | as BY4743; yal016w:: kanMX4 / YAL016W |
| TPD3 homozygous deletant | as BY4743; yal016w:: kanMX4 / yal016w:: kanMX4 |
| CCR4 heterozygous deletant | as BY4743; yal021c :: kanMX4 / YAL021C |
| CCR4 homozygous deletant | as BY4743; yal021c :: kanMX4 / yal021c :: kanMX4 |
| RAD61 heterozygous deletant | as BY4743; ydr014w :: kanMX4 / YDR014W |
| RAD61 homozygous deletant | as BY4743; ydr014w :: kanMX4 / ydr014w :: kanMX4 |
| STP22 heterozygous deletant | as BY4743; ycl008c :: kanMX4 / YCL008C |
| STP22 homozygous deletant | as BY4743; ycl008c :: kanMX4 / ycl008c :: kanMX4 |
| RTP2 heterozygous deletant | as BY4743; ydl007w :: kanMX4 / YDL007W |
| GPD1 heterozygous deletant | as BY4743; ydl022w :: kanMX4 / YDL022W |
| GPD1 homozygous deletant | as BY4743; ydl022w :: kanMX4 / ydl022w :: kanMX4 |
| IDP1 heterozygous deletant | as BY4743; ydl066w :: kanMX4 / YDL066W |
| IDP1 homozygous deletant | as BY4743; ydl066w :: kanMX4 / ydl066w :: kanMX4 |
| HO heterozygous deletant | as BY4743; ydl227c :: kanMX4 / YDL227C |
| CDC34 heterozygous deletant | as BY4743; ydr054c :: kanMX4 / YDR054C |
| NBP2 heterozygous deletant | as BY4743; ydr162c :: kanMX4 / YDR162C |
| NBP2 homozygous deletant | as BY4743; ydr162c :: kanMX4 / ydr162c :: kanMX4 |
| RAD9 heterozygous deletant | as BY4743; ydr217c :: kanMX4 / YDR217C |
| RAD9 homozygous deletant | as BY4743; ydr217c :: kanMX4 / ydr217c :: kanMX4 |
| YCG1 heterozygous deletant | as BY4743; ydr325w :: kanMX4 / YDR325W |
| MUS81 heterozygous deletant | as BY4743; ydr386w :: kanMX4 / YDR386W |
| MUS81 homozygous deletant | as BY4743; ydr386w :: kanMX4 / ydr386w:: kanMX4 |
| SMC2 heterozygous deletant | as BY4743; yfr031c :: kanMX4 / YFR031C |
| MAD1 heterozygous deletant | as BY4743; ygl086w :: kanMX4 / YGL086W |
| MAD1 homozygous deletant | as BY4743; ygl086w :: kanMX4 / ygl086w :: kanMX4 |
| RAD54 heterozygous deletant | as BY4743; ygl163c :: kanMX4 / YGL163C |
| RAD54 homozygous deletant | as BY4743; ygl163c :: kanMX4 / ygl163c :: kanMX4 |
| DBF2 heterozygous deletant | as BY4743; ygr092w :: kanMX4 / YGR092W |
| DBF2 homozygous deletant | as BY4743; ygr092w:: kanMX4 / ygr092w:: kanMX4 |
| CLB1 heterozygous deletant | as BY4743; ygr108w :: kanMX4 / YGR108W |
| CLB6 heterozygous deletant | as BY4743; ygr109c :: kanMX4 / YGR109C |
| CTF8 heterozygous deletant | as BY4743; yhr191c :: kanMX4 / YHR191C |
| CTF8 homozygous deletant | as BY4743; yhr191c :: kanMX4 / yhr191c :: kanMX4 |
| SMC3 heterozygous deletant | as BY4743; yjl074c :: kanMX4 / YJL074C |
| PBS2 heterozygous deletant | as BY4743; yjl128c :: kanMX4 / YJL128C |
| PBS2 homozygous deletant | as BY4743; yjl128c :: kanMX4 / yjl128c :: kanMX4 |
| HSL1 heterozygous deletant | as BY4743; ykl101w :: kanMX4 / YKL101W |
| HSL1 homozygous deletant | as BY4743; ykl101w :: kanMX4 / ykl101w :: kanMX4 |
| MEU1 heterozygous deletant | as BY4743; ylr017w :: kanMX4 / YLR017W |
| MEU1 homozygous deletant | as BY4743; ylr017w :: kanMX4 / ylr017w :: kanMX4 |
| PNP1 heterozygous deletant | as BY4743; ylr209c :: kanMX4 / YLR209C |
| PNP1 homozygous deletant | as BY4743; ylr209c :: kanMX4 / ylr209c :: kanMX4 |
| CLB4 heterozygous deletant | as BY4743; ylr210w :: kanMX4 / YLR210W |
| YCS4 heterozygous deletant | as BY4743; ylr272c :: kanMX4 / YLR272C |
| MSN2 heterozygous deletant | as BY4743; ymr037c :: kanMX4 / YMR037C |
| MSN2 homozygous deletant | as BY4743; ymr037c :: kanMX4 / ymr037c :: kanMX4 |
| BUB2 heterozygous deletant | as BY4743; ymr055c :: kanMX4 / YMR055C |
| BUB2 homozygous deletant | as BY4743; ymr055c :: kanMX4 / ymr055c :: kanMX4 |
| UBX4 heterozygous deletant | as BY4743; ymr067c :: kanMX4 / YMR067C |
| UBX4 homozygous deletant | as BY4743; ymr067c :: kanMX4 / ymr067c :: kanMX4 |
| RIM11 heterozygous deletant | as BY4743; ymr139w :: kanMX4 / YMR139W |
| RIM11 homozygous deletant | as BY4743; ymr139w :: kanMX4 / ymr139w :: kanMX4 |
| MLH1 heterozygous deletant | as BY4743; ymr167w :: kanMX4 / YMR167W |
| MLH1 homozygous deletant | as BY4743; ymr167w :: kanMX4 / ymr167w :: kanMX4 |
| TOP2 heterozygous deletant | as BY4743; ynl088w :: kanMX4 / YNL088W |
| RPD3 heterozygous deletant | as BY4743; ynl330c :: kanMX4 / YNL330C |
| DNL4 heterozygous deletant | as BY4743; yor005c :: kanMX4 / YOR005C |
| DNL4 homozygous deletant | as BY4743; yor005c:: kanMX4 / yor005c:: kanMX4 |
| HIS3 heterozygous deletant | as BY4743; yor202w :: kanMX4 / YOR202W |
| HIS3 homozygous deletant | as BY4743; YOR202W :: kanMX4 / YOR202W :: kanMX4 |
| HRK1 heterozygous deletant | as BY4743; YOR267C :: kanMX4 / YOR267C |
| HRK1 homozygous deletant | as BY4743; YOR267C:: kanMX4 / YOR267C:: kanMX4 |
| MET7 heterozygous deletant | as BY4743; YOR241W :: kanMX4 / YOR241W |
| MET7 homozygous deletant | as BY4743; YOR241W:: kanMX4 / YOR241W:: kanMX4 |
| TUM1 heterozygous deletant | as BY4743; YOR251C :: kanMX4 / YOR251C |
| TUM1 homozygous deletant | as BY4743; YOR251C:: kanMX4 / YOR251C:: kanMX4 |
| RAD1 heterozygous deletant | as BY4743; YPL022W :: kanMX4 / YPL022W |
| RAD1 homozygous deletant | as BY4743; YPL022W:: kanMX4 / YPL022W:: kanMX4 |
| TPO3 heterozygous deletant | as BY4743; YPR156C :: kanMX4 / YPR156C |
| TPO3 homozygous deletant | as BY4743; YPR156C:: kanMX4 / YPR156C:: kanMX4 |
| CLN2 heterozygous deletant | as BY4743; YPL256C :: kanMX4 / YPL256C |
| CLB5 heterozygous deletant | as BY4743; YPR120C :: kanMX4 / YPR120C |

**Additional Table 6:** Yeast strains used in this study.
